# Injuries Sustained With Falls From Height in Crossing the United States-Mexico Border at a Level I Trauma Center: A Prospective Cohort Study

**DOI:** 10.5435/JAAOSGlobal-D-23-00005

**Published:** 2023-06-07

**Authors:** Michael M. Polmear, Tyler C. Nicholson, James A. Blair, Ahmed M. Thabet, Adam H. Adler, Rajiv Rajani

**Affiliations:** From theDepartment of Orthopaedic Surgery, Texas Tech University Health Sciences Center El Paso, El Paso, Texas (Dr. Polmear, Dr. Nicholson, Dr. Thabet, Dr. Adler, and Dr. Rajani); the Department of Orthopaedic Surgery, Medical College of Georgia at Augusta University, Augusta, GA (Dr. Blair).

## Abstract

The US Department of Homeland Security has reported increases in encounters and apprehensions at the US Southwest border for the past several years. The purposes of this study were to assess the demographics, patterns of injuries, and surgical interventions, associated with falls from height along the US-Mexico border.

**Methods:**

A prospective cohort study was conducted at a Level I trauma center from January 2016 through December 2021 of all patients who fell from height crossing the US-Mexico border and presented with injuries requiring admission.

**Results:**

A total of 448 patients were admitted with a median age of 30 years (interquartile range [IQR] 16, range 6 to 65). Monthly frequency of admissions increased markedly with a median of 18.5 (IQR 5.3) in 2021. Patients presented with limited health data, and comorbidities were identified in 111 patients (24.7%). Median height fallen was 5.5 m (18 ft). Patients sustaining a fall from ≥ 5.5 m were markedly more likely to have an Injury Severity Score (ISS) of > 15. Median length of stay was 9 days (IQR 11). There were a total of 1,066 injuries with 723 extremity and pelvic; 236 spine; and 107 head or neck, face, thorax, or abdominal injuries. Median ISS was 9.0 (IQR 7, range 1 to 75, 33% > 15). Tibial plafond fracture and spine injury were markedly associated with longer lengths of stay and ISS > 15. All injuries resulted in 635 separate surgical events and 930 procedures. Clinical follow-up occurred in 55 patients (12.2%), with median duration of 28 days (range 6 days to 8 months).

**Discussion:**

Injuries associated with border crossings and falls from height were serious and increased in frequency. As the US policy on border security evolves, surgeons in these regions should be prepared to handle the associated injuries and sequelae. Prevention of these serious and debilitating injuries should be undertaken to decrease the burden of disease.

The US-Mexico border spans 3,187 km (1,980 miles) from San Diego, California, to Brownsville, Texas. US Border Patrol (USBP) agents apprehended over two million individuals crossing outside of legal ports of entry in fiscal year (FY) 2022,^1^ a 3-fold increase over the past 5 years. The El Paso component oversees 13% of the linear border (425 km, 264 miles) and apprehended 258,766 individuals (13% of all Southwest apprehensions) in FY2022.^2^ Crossing outside of legal points of entry is hazardous with harsh terrain and a physical constructed barrier that reaches 1.8 to 9.1 m (6 to 30 ft) in approximately one-third of the border (1,046 km, 650 miles), primarily built after the 2006 Secure Fence Act.^3^ In some sections, the height of the border barrier increased with enactment of the Border Security and Immigration Enforcement Improvements Executive Order signed on January 24, 2017.^4^ This resulted in 406 miles of existing 1.8 to 5.2-m (6 to 17-ft) link fence replaced with 5.5 to 9.1-m (18 to 30-ft) steel bollard barrier, in addition to 79 km (49 miles) of a new bollard barrier. USBP conducts in-person and electronic surveillance along the border.^5^ Migrants sustaining injuries while traversing the border are assessed and transferred to a hospital under USBP custody.

Recent literature exploring the healthcare implications associated with immigration has identified the increasing trend,^6-8^ cost,^8^ breadth of injuries,^6^ high incidence of musculoskeletal injuries,^9^ coincidence of spine injuries,^10^ medical complications,^11^ potential for mortality,^12^ incidence of neurotrauma,^13^ and severity of injury.^14^ The association of injury severity and recent barrier height increase was proposed by Liepert et al. in San Diego, California,^14^ and the present study expanded on this finding in another location and with specific injury characteristics contributing to overall severity.

There was one primary and two secondary hypotheses: First, as the barrier height increased, there would be a higher incidence of ISS > 15, typified by multiply injured patients sustaining tibial plafond and spine fractures with longer hospital length of stay (LOS). Second, musculoskeletal injuries would constitute most injuries, constitute most surgical interventions, and markedly contribute to injury severity trauma scoring. Finally, clinical follow-up would be hampered by several barriers, including variable disposition.

## Methods

A prospective cohort study was conducted from January 2016 to December 2021 for all patients admitted for injuries sustained with falls from height at the US-Mexico border. Institutional review board approval was obtained. The institution is situated on the US-Mexico border and is the only ACS-verified Level I trauma and referral center for over 450 km (280 miles) from New Mexico to the West Texas border.

All patients who were admitted under custody of USBP after sustaining injuries in traversing the border barrier were included. Patients presenting under USBP custody with injuries sustained by other mechanisms after traversing the border were excluded, including 50 patients with motor vehicle accidents, pedestrian accidents from roadway crossings, exposure, animal bites, and ballistic injuries. Patients presenting with histories and injuries concerning for falls from the border barrier and not in USBP custody were excluded because of the absence of corroboration of the distance fallen and to minimize heterogeneity. Reporting was conducted based on the Strengthening the Reporting of Observational Studies in Epidemiology (STROBE) statement and the REporting of studies Conducted using Observational Routinely collected health Data (RECORD) extension statement.^15^

Collected data included demographic parameters, mechanism of injury, height fallen based on the height of the structure at the site of injury, LOS, ICU admissions, injury characteristics, procedures conducted, and clinical follow-up assessments. ISS^16^ and New Injury Severity Score (NISS)^17^ were calculated from the Abbreviated Injury Scale (AIS) scores for each body region. Similarly, the Orthopaedic Injury Severity Score (OISS), proposed here, was calculated as the sum of squares of the three most severe extremity and pelvic injuries to account for the range of musculoskeletal injuries and bilateral extremity injuries. Injuries were categorized based on the ISS six body regions of the head, neck, and cervical spine; face; chest and thoracic spine; abdomen and lumbar spine; upper extremities, lower extremities, and pelvic skeleton; and external.^16^

## Statistical Analysis

Calculations were done using SAS software (Version 9.2; SAS Institute). Normality was assessed using the Shapiro-Wilk expanded test. Continuous variables were described using median and interquartile range (IQR), and categorical variables were described using frequency and percentage. The Mann-Whitney *U* test compared medians between groups of continuous nonparametric data.^18^ Relative risk (RR) and 95% confidence intervals (CIs) were calculated for binary outcome cohort comparisons. Linear regression analysis was conducted to assess correlation. An alpha value of < 0.05 or with Bonferroni correction for multiple comparisons was considered significant.

## Results

### Demographics

**Figure 1:**
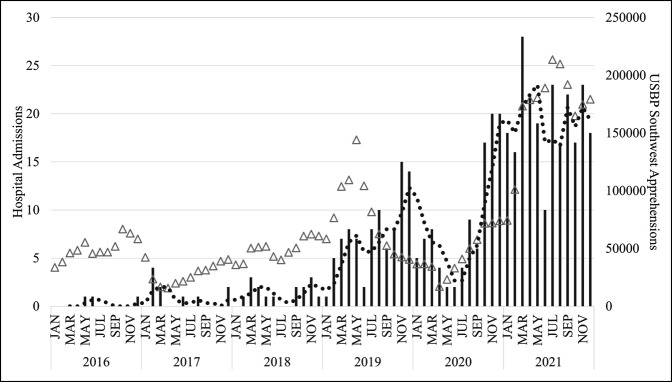
Graph showing monthly admissions for patients sustaining injuries from the US-Mexico international border fence and bridge included in the study (N = 448, bars, primary axis) and Southwest US Border Patrol (USBP) apprehensions (triangles, secondary axis). Solid circular markers are a 3-month moving average for admissions. Simple linear regression was used to test whether month of study significantly predicted the number of hospital admissions. The fitted regression model was as follows: Admission count = 0.3 [month]—4.6. The overall regression was statistically significant: R2 = 0.671, F(1, 70) = 143.117, *P* < 0.001. Simple linear regression was used to test whether USBP apprehensions significantly predicted the number of hospital admissions. The fitted regression model was as follows: Admission count = 0.000259[apprehensions] + 18.4. The overall regression was statistically significant: R2 = 0.419, F(1, 70) = 50.417, *P* < 0.001.

A total of 498 patients were admitted; 448 (90%) met inclusion criteria with a median age of 30 years (IQR 16, range 6 to 65); and 52% were male (Table 1). Monthly frequency of admissions increased significantly with a median of 0 (IQR 0.25) in 2016 to 18.5 (IQR 5.3) in 2021 (*P* < 0.001). USBP Southwest region overall apprehensions were significantly correlated with hospital admissions (*P* < 0.001), with zero to four patients admitted to the hospital monthly for every 10,000 apprehensions (Figure 1). All patients identified as Hispanic or Latino from 13 countries in Central and South America, except for one patient from China. There were a total of 1066 injuries with 723 extremity and pelvic (105 upper extremity, 549 lower, and 69 pelvic or acetabular); 236 spine; and 107 head/neck, face, thorax, or abdominal injuries for a median of two injuries per patient (range 1 to 11). All patients were admitted for observation (N = 34, 7.6%) or surgical treatment (N = 414, 92.4%). Two hundred forty one patients (53.8%) were admitted to the ICU for a median of 3 days (IQR 2), and the overall median LOS for all patients was 9 days (IQR 11).

**Table 1:** Demographics and Injury Characteristics

Variable	Total (N)	Total (%)	Median	IQR	Range
Patients	448	100	—	—	—
Age	448	100	30	16	6-65
Sex (male)	233	52.0	—	—	—
Body mass index (kg/m^2^)	448	100	28	7.6	13.3-45
Comorbidities	111	24.8	0	0	0-3
Distance fall (m)	448	100	5.5	1.5	0.9-15.2
Hospital LOS (d)	448	100	9.0	11.0	1-43
ICU LOS (d)	241	53.8	3	2	1-23
Injuries					
*Extremity and Pelvis Injuries*	436 patients	97.3	1	1	0-5
Upper	105	14.4	0	0	0-5
Lower	449	76.1	1	1	0-5
Pelvic	69	9.6	0	0	0-3
Subtotal	723	67.8	2	2	1-11
*Spine Injuries*	236 (117 patients)	23.2 (26.2)	0	1	0-7
*Head, Neck, and Face*					
*Thorax and Abdominal Injuries*	107 (60 patients)	10.0 (13.4)	0	0	0-6
*Total Injuries*	1066	100	2	2	1-11
Abbreviated Injury Scale Scores					
Head, neck, and C-spine	31	6.9	0	0	0-6
Face	20	4.5	0	0	0-4
Chest, diaphragm, and T-spine	41	9.2	0	0	0-6
Abdomen and L-Spine	107	23.9	0	0	0-4
Extremities and pelvis	436	97.3	3	1	0-4
External	1	0.2	0	0	0-2
Severity scores					
ISS	448	100	9.0^a^	7.0	1-75
NISS	448	100	13.0^b^	13.0	1-88
OISS	448	100	9.0^c^	11.3	0-36
Surgical cases					
Extremity/Pelvic cases	578 cases 873 procedures	91.0	1.0	1.0	0-3
Spine cases	40	6.3	0.0	0.0	0-2
Head, neck, face, thorax, and abdominal cases	17	2.7	0.0	0.0	0-2
Total cases	635 930 procedures (414 patients)	100 (92.4)	1.0	1.0	0-4
Complications	7 patients	1.6	0	0	0-3
Return to OR	4	0.9	0	0	0-1
Clinic follow-up	55	12.2%	28 d	6 d	8 mo
Definitive surgery done	414	92.4%	—	—	—
Additional surgery recommended	34	7.6%	—	—	—

ISS = Injury Severity Score, LOS = length of stay, OISS = Orthopaedic Severity Score, OR = operating room, NISS = New Injury Severity Score

a Mann-Whitney *U* Test for NISS vs. ISS, *P*-value 3.00E-07.

b Mann-Whitney *U* Test for OISS vs. ISS, *P*-value 0.0983.

c Mann-Whitney *U* Test for OISS vs. NISS, *P*-value 1.066E-11.

Simple linear regression was used to test whether the height of fall significantly predicted the Injury Severity Score. The fitted regression model was as follows: Injury Severity Score = 0.89[height (m)] + 8.7. The overall regression was statistically significant: R2 = 0.0340, F(1, 291) = 10.2, *P* = 0.00152.

Simple linear regression was used to test whether the presence of tibial plafond fracture significantly predicted the Injury Severity Score. The fitted regression model was as follows: Injury Severity Score = 1.6[tibial plafond fracture (n)] + 11.8. The overall regression was statistically significant: R2 = 0.00982, F(1, 446) = 4.4, *P* = 0.0360.

Simple linear regression was used to test whether the presence of tibial plafond fracture significantly predicted the hospital length of stay. The fitted regression model was as follows: Length of stay = 5.1[tibial plafond fracture (n)] + 9.1. The overall regression was statistically significant: R2 = 0.114, F(1, 341) = 43.9, *P* < 0.001.

Patients presented with limited health data, and comorbidities were identified in 111 patients (24.7%), including tobacco use (8.2%), COVID-19 (6.9%), hypertension (5.3%), diabetes mellitus II (3.3%), and pregnancy (1.7%) (Supplemental Data, http://links.lww.com/JG9/A285). Hospital LOS was significantly longer for patients presenting with comorbidities (11, IQR 12 vs. 8, IQR 9.5 days, *P* < 0.001). Patients presenting with COVID-19 had significantly longer LOS (14, IQR 9 vs. 8, IQR 10 days, *P* < 0.001).

There were 723 musculoskeletal injuries, which constituted the majority (67.8%) of all injuries in 436 patients (97.3%) and 578 (91.0%) of all surgical interventions. Multiple musculoskeletal injuries were common, with two occurring in 188 (42.0%), three in 67 (15.0%), four in 23 (5.1%), and five in 9 (2.0%) patients.

### Extremity, Pelvic Ring, and Acetabular Injuries

There were 105 upper extremity injuries (14.4% of all extremity/pelvic injuries). There were 33 (31.2%) soft-tissue wounds and 19 (18.2%) open fractures. A total of 41 (39.4%) upper extremity injures required surgical intervention. Distal radius fractures constituted the most common injury (N = 15, 14.4% of all upper extremity injuries), and 11 (73.3%) required surgical intervention. The 14 most common injuries with at least three occurrences constituting 66.3% of upper extremity injuries are presented in Table 2 with all injuries tabulated in Supplemental Data, http://links.lww.com/JG9/A285.

**Table 2:** Most Common Upper Extremity Orthopaedic Injuries

Injury	N	Open (N, %)	Gustilo-Anderson (N, %)			Surgical (N, %)	Location (%)	Total^a^ (%)
			1	2	3A			
Distal radius fx	15	2 (13.3)	1 (6.6)	1 (6.6)	0 (0)	11 (73.3)	14.4	2.1
Hand laceration	6	6 (100)	0 (0)	0 (0)	0 (0)	3 (50)	5.8	0.8
Scaphoid fx	6	1 (16.6)	0 (0)	0 (0)	0 (0)	4 (66.6)	5.8	0.8
Elbow terrible triad	5	1 (20)	1 (20)	0 (0)	0 (0)	5 (100)	4.8	0.7
Radial head fx	5	1 (20)	0 (0)	1 (20)	0 (0)	3 (60)	4.8	0.7
Humeral distal fx	4	3 (75)	0 (0)	2 (50)	1 (25)	4 (100)	3.8	0.6
Monteggia fx	4	3 (75)	3 (75)	0 (0)	0 (0)	4 (100)	3.8	0.6
Perilunate dislocation	4	0 (0)	0 (0)	0 (0)	0 (0)	4 (100)	3.8	0.6
Ulnar diaphyseal fx	4	1 (25)	1 (25)	0 (0)	0 (0)	3 (75)	3.8	0.6
Ulnar styloid fx	4	0 (0)	0 (0)	0 (0)	0 (0)	0 (0)	3.8	0.6
Phalanx fx	3	1 (33.3)	0 (0)	0 (0)	1 (33.3)	2 (66.6)	2.9	0.4
Distal radius and ulna fx	3	3 (100)	2 (66.6)	1 (33.3)	0 (0)	3 (100)	2.9	0.4
Radial neck fx	3	0 (0)	0 (0)	0 (0)	0 (0)	3 (100)	2.9	0.4
Shoulder dislocation	3	0 (0)	0 (0)	0 (0)	0 (0)	0 (0)	2.9	0.4
*Subtotal*	*69*	*22 (21.2)*	*14 (13.5)*	*5 (4.8)*	*2 (1.9)*	*49 (47.1)*	*66.3*	*9.5*
Total	105	33 (31.7)	10 (9.6)	5 (4.8)	4 (3.8)	41 (39.4)	100	14.4
Median (IQR)	2 (2)	0 (1)	0 (0)	0 (0)	0 (0)	1 (3)	1.9 (2)	0.3 (0.3)
		0 (100)	0 (0)	0 (0)	0 (0)	100 (100)		
Range	1-15	0-6	0-3	0-2	0-2	0-11	1-14	0.1-2.1
		(0-100)	(0-100)	(0-50)	(0-100)	(0-100)		

fx = fracture, IQR = interquartile range

a Total extremity and pelvic ring and acetabular injuries.

There were 549 lower extremity injuries (76.1% of all extremity/pelvic injuries). There were 128 (23.3%) soft-tissue injuries and 98 (17.8%) open fractures. A total of 424 (77.1%) lower extremity injuries required surgical intervention. Tibial plafond fractures constituted the most common injuries with 137 fractures (N = 122 patients, 15 bilateral, 24.9% of all lower extremity injuries, 99.3% treated surgically). There were 38 (27.7%) open fractures with 14 (10.9%) Gustilo-Anderson IIIA severity. Patients with a tibial plafond fracture had significantly longer LOS (median 14, IQR 13 days vs. 7, IQR 4, *P* < 0.001), greater ISS (median 13, IQR 7 vs. 9, IQR 12, *P* = 0.0164), and greater RR of ISS > 15 (RR 1.42, 95% CI 1.09 to 1.84, *P* = 0.0101). Distance fallen was not associated with the incidence of tibial plafond fracture (median 6.1, IQR 1.2 vs. median 5.5, IQR 1.5 m for patients without tibial plafond, *P* = 0.20). The 13 most common injuries with at least seven occurrences constituting 92.2% of lower extremity injuries are presented in Table 3 with all injuries tabulated in Supplemental Data, http://links.lww.com/JG9/A285.

**Table 3:** Most Common Lower Extremity Orthopaedic Injuries

Injury	N	Open (N, %)	Gustilo-Anderson (N, %)						Surgical (N, %)	Location (%)	Total^a^ (%)
			1	2	3	3A	3B	3C			
Tibial plafond fx	137	38 (27.7)	7 (5.1)	12 (8.7)	18 (13.1)	15 (10.9)	2 (1.4)	1 (0.7)	137 (100)	25.0	19.0
Ankle fx	73	7 (9.5)	0 (0)	3 (4.1)	4 (5.4)	4 (5.4)	0 (0)	0 (0)	62 (84.9)	13.3	10.1
Calcaneus fx	62	5 (8)	2 (3.2)	2 (3.2)	1 (1.6)	1 (1.6)	0 (0)	0 (0)	15 (24.1)	11.3	8.6
Tibial diaphysis fx	51	26 (50.9)	4 (7.8)	18 (35.2)	3 (5.8)	3 (5.8)	0 (0)	0 (0)	51 (100)	9.3	7.1
Talus fx	47	6 (12.7)	1 (2.1)	4 (8.5)	1 (2.1)	1 (2.1)	0 (0)	0 (0)	23 (48.9)	8.6	6.5
Tibial plateau fx	36	5 (13.8)	2 (5.5)	2 (5.5)	1 (2.7)	1 (2.7)	0 (0)	0 (0)	33 (91.6)	6.6	5.0
Proximal femur fx	24	1 (4.1)	0 (0)	0 (0)	1 (4.1)	1 (4.1)	0 (0)	0 (0)	22 (91.6)	4.4	3.3
Patella fx	20	8 (40)	3 (15)	3 (15)	2 (10)	2 (10)	0 (0)	0 (0)	18 (90)	3.6	2.8
Midfoot fx	19	0 (0)	0 (0)	0 (0)	0 (0)	0 (0)	0 (0)	0 (0)	10 (52.6)	3.5	2.6
Femoral diaphysis fx	13	1 (7.6)	0 (0)	0 (0)	1 (7.6)	1 (7.6)	0 (0)	0 (0)	13 (100)	2.4	1.8
Forefoot fx	9	0 (0)	0 (0)	0 (0)	0 (0)	0 (0)	0 (0)	0 (0)	0 (0)	1.6	1.2
Leg laceration	8	8 (100)	0 (0)	0 (0)	0 (0)	0 (0)	0 (0)	0 (0)	5 (62.5)	1.5	1.1
Knee traumatic arthrotomy	7	7 (100)	0 (0)	0 (0)	0 (0)	0 (0)	0 (0)	0 (0)	7 (100)	1.3	1.0
*Subtotal*	*506*	*112 (20.4)*	*19 (3.5)*	*44 (8.0)*	*32 (5.8)*	*29 (5.3)*	*2 (0.4)*	*1 (0.2)*	*396 (72.0)*	*92.2*	*70.1*
Total	549	128 (23.3)	19 (3.5)	45 (8.2)	34 (6.2)	32 (5.8)	2 (0.4)	1 (0.2)	424 (77.1)	100	76.1
Median (IQR)	3 (13.5)	1 (5)	0 (0)	0 (0)	0 (1)	0 (1)	0 (0)	0 (0)	1 (9.8)	0.5 (2.5)	0.4 (1.9)
		8.8 (75)	0 (0)	0 (0)	0 (2.3)	0 (2.3)	0 (0)	0 (0)			
Range	1-137	0-37	0-7	0-18	0-17	0-15	0-2	0-1	0-137	0.2-25	0.14-18.9
		(0-100)	(0-15)	(0-35)	(0-100)	(0-100)	(0-1.5)	(0-0.7)	(0-100)		

fx = fracture, IQR = interquartile range

a Total extremity and pelvic ring and acetabular injuries.

There were 69 patients with pelvic or acetabular fractures (15.4% of all patients, 10% of all extremity/pelvic injuries). There were no open injuries, and 28 (40.6%) required surgical intervention (Supplemental Data, http://links.lww.com/JG9/A285). There were 42 pelvic ring fractures (61% of all pelvic injuries), and 21 (50.0%) required surgical intervention because of Denis Zone 2 or 3 involvement, failed trial of mobilization, or abnormal examination under anesthesia.^19^ Pelvic ring injuries occurred in 14 injury types with Young-Burgess lateral compression I, incomplete Denis Zone 1 sacral fracture being the most common with 15 injuries (35.7%) and a 13% surgical rate (Supplemental Data, http://links.lww.com/JG9/A285).

### Spine Injuries

A total of 117 patients (26.2%) sustained spine injuries, and 39 patients underwent surgical treatment, constituting a 33.3% surgical rate for patients with spine injuries and 8.7% for all patients (Supplemental Data, http://links.lww.com/JG9/A285). There were 152 spine injuries within four distinct regions (ie cervical, thoracic [T1-T9], thoracolumbar [T10-L2], and lumbar [L3-5]); 25.6% were treated surgically overall, with the majority (57.2%) occurring at the thoracolumbar level where 34.5% of thoracolumbar injuries were treated surgically (Supplemental Data, http://links.lww.com/JG9/A285). In patients with spine injuries, rates of associated injuries were 17.1% upper extremity, 70.1% lower extremity, and 27.4%. pelvic ring and acetabular fractures. Patients with a spine injury had significantly higher fall (median 6, IQR 5 vs. 5, IQR 1.5 m, *P* < 0.001), longer LOS (median 20, IQR 11 vs. 9, IQR 5 days, *P* < 0.001), greater ISS (median 11, IQR 10 vs. 8, IQR 8, *P* < 0.001), and greater RR of ISS > 15 (RR 3.23, 95% CI 2.53 to 4.12, *P* < 0.001).

### Head, Neck, Face, Thorax, and Abdominal Injuries

A total of 60 patients (13.4%) sustained 107 head, neck, face, thorax, or abdominal injuries. Fifteen patients (25%) were treated surgically (Supplemental Data, http://links.lww.com/JG9/A285). Traumatic brain injury with intracranial hemorrhage was the most common head and neck injury and occurred in 19 patients (48.7% of head and neck injuries), and four (21%) were treated surgically. Facial fracture and laceration were the most common face injuries and occurred in 11 and nine patients, respectively (44 and 36% of face injuries, respectively), and three patients (15%) were treated surgically. Rib fractures were the most common thoracic injury and occurred in 11 patients (37.9% of thoracic injuries) and were all treated nonsurgically. No other thoracic injuries were treated surgically. Renal and hepatic lacerations were the most common abdominal injuries and occurred in four and three patients, respectively (28.5 and 21.4% of abdominal injuries, respectively), and were all treated nonsurgically.

### Complications

Seven patients (1.6%) had a total of 10 complications (2.0% incidence) during hospitalization (Table 4). There were four unexpected returns to the operating room, including one for iatrogenic bladder injury, revision lumbosacral fusion, revision posterior pelvic fixation, and revision mandibular fracture fixation. One patient had a spontaneous abortion from her injury requiring dilatation and curettage of products of conception. One patient presented with traumatic brain injury with diffuse axonal injury, underwent tracheotomy, and died from cardiac arrest. There were no other known mortalities.

**Table 4:** Complications

Patient	Complications	Additional Surgery
1	Iatrogenic bladder injury during posterior pelvic fixation	Bladder repair
2	L2-S1 interbody malalignment during posterior fusion	Revision L2-S1 interbody posterior fusion
3	Iatrogenic L5 nerve palsy during posterior pelvic fixation	Revision posterior pelvic fixation
4	Mandibular malreduction	Revision mandible ORIF
5	Cardiac arrest<	Tracheostomy
	Deceased—organ donation	
6	Spontaneous abortion because of injury	Dilatation and curettage of products of conception
7	Neuroleptic malignant syndrome	—
	DVT	
	Readmission	
Total (% all patients)	7 patients (1.6)	6 (1.3)
	10 complications (2.0)	

ORIF: open reduction and internal fixation

### Injury Severity Scores

Median ISS was 9 (IQR 7, range 1 to 75), and median NISS was 13 (IQR 13, range 1 to 88). Height of fall was a significant predictor of increasing ISS (*P* = 0.00152). Thirty-three percent of patients had ISS > 15 and NISS > 17. Median OISS was 9 (IQR 11, range 0 to 36). NISS was significantly greater than ISS (*P* < 0.001) and OISS (*P* < 0.001, Bonferroni correction 0.0166). ISS and OISS were not significantly different (*P* = 0.10). The reported median height at the scene of injury was 5.5 m (IQR 1.5, range 0.9 to 15.2). Maximum fallen distance occurred where the border barrier abutted an overpass bridge. Patients sustaining a fall from ≥ 5.5 m were significantly more likely to have an ISS of > 15 (RR 3.14, 95% CI 2.27 to 4.35, *P* < 0.001). RR of ISS > 15 increased significantly in October 2020 after majority completion of the bollard barrier wall with height reaching 9.1 m in El Paso, Texas (RR 1.35, 95% CI 1.01 to 1.80, *P* = 0.0406). Female patients had a significantly higher RR of ISS > 15 than male patients (RR 1.48, 95% CI 1.13 to 1.93, *P* = 0.00400).

### Surgical Intervention

A total of 635 separate surgical events and 930 procedures in 414 patients (92.4%) were done, with each patient undergoing a median of one surgical event (IQR 1, range 0 to 4). Extremity or pelvic surgery was done on 386 patients (86.1%) a median of one time (IQR 1, range 0 to 3); spine surgery on 39 patients (once each, except for a single revision surgery); and head, neck, face, thorax, or abdominal surgery on 15 patients (3.3%). Patients with open tibial plafond fractures had significantly more median procedures than patients with closed tibial plafond fractures (N = 39, median 3, IQR 1, range 1 to 4 vs. N = 83, median 2, IQR 1, range 0 to 2, *P* < 0.001).

### Clinical Follow-Up

Fifty-five patients (12.2%) had median clinic follow-up duration of 28 days (IQR 36 days, range 6 days to 8 months) in affiliated local clinics. Patients seen in clinic were pending transfer disposition or were staying with local family members. Barriers to care included access to durable medical equipment (DME) for adherence to weight-bearing restrictions, discharge instruction translation into native language, difficulty with negative pressure wound therapy (NPWT) equipment, suture and staple removal with wound evaluation, transition from plaster splint to orthosis, and medication refill.

Definitive surgery was done in 414 patients (92.4%), and surgical consultation was recommended in the remaining 34 patients (7.6%) at the time of hospital discharge. Deportation, detention, asylum within the United States, and humanitarian release to family members within the United States were the most common dispositions.

## Discussion

Injuries sustained by traversing the US-Mexico border barrier occurred frequently, were often serious, and were dominated by musculoskeletal injuries, and 38% required multiple surgical interventions. The experience at a single trauma center along the US-Mexico border provided insight into the injury characteristics and aligned with reports of other trauma centers along the border.^6,7,9,10,20^ Male patients constituted 52% of the patients in this study, which was similar to some reports^13,20^ (47 to 56% male) but less than other reports (62 to 84% male),^6,7,10^ suggesting variable immigration patterns. Figure 1 demonstrated the variability in monthly admissions, relative nadir in the early COVID-19 pandemic in spring 2020, and notable correlation with monthly USBP apprehensions. Overall, there was a notable increase in hospital admissions, which corroborated other reports attributing the frequency to increasing migration^1^ and height of the border barrier posing a more dangerous obstacle.^14^ A previous study found a similar trend of increasing trauma center encounters for falls from the border barrier associated with increased barrier height in San Diego, California.^7^

Injury severity was markedly associated with barrier height at the scene of injury. RR of ISS > 15 was markedly greater for falls ≥ 5.5 m. Tibial plafond fracture was markedly associated with longer LOS, greater ISS, and ISS > 15, but was not associated with distance fallen because these fractures occurred between 1.5 and 9.1 m. Longer LOS for patients with tibial plafond fractures was attributed to soft-tissue monitoring for open injuries, being multiply injured, and limited options for interim discharge before definitive fixation. Spine injury was markedly associated with higher height fallen, longer LOS, greater ISS, and ISS > 15.

Musculoskeletal injuries, particularly lower extremity trauma, were the most common, aligned with previous reports, and partially confirmed the second hypothesis.^6,7,10,20^ Open fractures were observed at higher rates in the study of Burk et al. with 33% open ankle fractures (vs. 9.5%) and 44% tibial plafond fractures (vs. 27.7%).^20^ The median number of extremity and pelvic injuries in this study was 1 (IQR 1) with a range of 1 to 5, which was similar to a previous report of Burk et al. of 1.58 ± 0.07 fractures per patient. It was hypothesized that the OISS, represented by the sum of squares of the three greatest extremity and pelvic AIS scores, would be markedly higher than the ISS, which did not account for multiple injuries in the same body region. In this study, 42% of patients had at least two extremity and pelvic ring injuries. The median OISS and ISS were not markedly different, which was attributable to the median number of extremity and pelvic injuries of 1. However, the range of OISS was 0 to 36 with 31.8% > 15 and 18.0% > 17, demonstrating the severity of musculoskeletal injuries and comparison with severe ISS and NISS, respectively.

The incidence of spine injuries in this study was 26.2%, which was lower than a prior study assessing falls from height (83%),^21^ but was similar to previously reported rates found in patients who sustained falls from the border barrier (26.4%)^20^ and border bridges (27.8%).^10^ Differences in injury patterns arose from variability in height fallen based on the use of climbing aids (e.g. ropes, ladders).

The dispositions of patients varied, and definitive locations were often unknown, which was common among refugees and asylees seeking legal permanent residence.^22^ Most patients remained inpatient for observation, soft-tissue evaluation, and case management under USBP custody. Temporary release to local family members, nonprofit organizations, and detention centers between staged procedures became feasible in mid-2020. This was particularly spurred as the community adapted to influxes of injured immigrants and patients with COVID-19 who occupied hospital beds with markedly longer LOS. The high rate of definitive fixation in 92.4% of patients demonstrated coordination among case management, the Department of Homeland Security, and surgical teams. Definitive treatment was prioritized in most cases where transfer and final dispositions were unknown. Final dispositions were handled on case-by-case basis by USBP with channels for asylum with notice to appear, humanitarian release, voluntary return to the country of origin, and detainment.^23^

The overall follow-up was 12% in affiliated clinics despite the intention and instructions to follow all patients, which confirmed the third hypothesis and aligned with findings at other institutions.^9^ Policies related to detention and release of apprehended individuals were fluid because of changing governmental policy, the COVID-19 pandemic, and space available for local detention.^24,25^ Aftercare, definitive fixation, and complications were unknown in all other patients.

This study has multiple limitations. First, lack of follow-up underestimates the postsurgical complication profile and precluded the ability to obtain radiographic or functional outcomes. Second, the study included a single Level I trauma center with a catchment area encompassing approximately 13% of the US-Mexico border. There were lower acuity trauma centers that treated patients injured from traversing the border, thereby underestimating the actual frequency of hospital admission from the catchment area. Third, only patients admitted after falls from height and in USBP custody were included, and patients treated and discharged from the emergency department were excluded, underestimating overall healthcare encounters. Furthermore, patients who sustained injuries or environmental exposure while fleeing on foot or in motor vehicles were excluded, which was approximately 50% of border-crossing injuries requiring medical care.^6^ Finally, resource utilization and cost merit additional analysis because a previous study reported median costs to be $42,000 per patient (IQR $68,625, range $8000 to 470,000) admitted and treated surgically after sustaining injuries in traversing the border.^8^ This study elucidated an injury and treatment characterization at a Level I trauma center along the US-Mexico border during a 6-year period that encompassed changes in border barrier.

Clinical implications of treating displaced patients with unknown access to surgical follow-up elucidated additional recommendations.

- Conduct surgical fixation and coverage or define a definitive treatment plan as soon as possible to avoid potential complications arising from staged treatment because of dispositions to another healthcare facility or to deportation.
- For patients requiring prolonged hospitalization, provide documentation for the medical and surgical reasons, such as compartment monitoring, pain control, medical management, anticipated surgeries for multisystem injuries, soft-tissue amenability for staged procedures, access to NPWT equipment, and wound healing for open fractures, to facilitate case management coordination with USBP for the final transfer date.
- Consider reliability of community and detention resources for holding to reduce inpatient census between staged procedures.
- Assume that once a patient is discharged, they will not be able to follow up in a clinic within the United States because some patients were deported or elected for voluntary return to the country of origin because of increased social support.
- Consider the use of absorbable sutures and skin closure techniques that do not require the removal of staples or sutures.
- Be cautious with multistage procedures, such as definitive fracture treatment in a uniplanar or circular external fixator, anticipated need for bone grafting, and bone transport, if patient discharge is going to be attempted. The decision to proceed with open reduction and internal fixation at the initial facility versus discharge with spanning external fixation merits individual considerations depending on the patient's disposition from case management and the Department of Homeland Security. Discharging to the country of origin in spanning external fixation, hastening definitive surgical incisions before soft-tissue rest, or delaying definitive fixation and necessitating extensive dissection and débridement of callus are suboptimal. However, patients with known dispositions and time lines with definitive surgical care access within the United States may benefit from treatment at the receiving facility to manage possible complications.
- Provide patients with detailed verbal and written instructions defining weight-bearing status, range-of-motion and activity restrictions, physical therapy instructions, signs and symptoms of infections, wound care instructions, and individualized summaries of care and surgical reports in native language to optimize outcomes and mitigate the problems associated with lack of follow-up.
- Provide patients with discharge medications because of limited pharmacy access, DME to facilitate weight-bearing restrictions, anticipated orthosis if plaster splint removal occurs in transit, and dressing materials.
- Recognize that patient circumstances may not allow for certain weight-bearing restrictions, such as non–weight-bearing of a lower extremity.

## Conclusion

Individuals who crossed into the United States and sustained injuries with falls from height presented with unique care challenges. This cohort sustained serious injuries with median AIS 3 (IQR 1, range 1 to 6) and had 16% open fracture, 19% tibial plafond fracture, and 26% spine injury with a 33% surgical rate. Polytrauma occurred in 33% of patients (ISS > 15). Multiple surgical interventions were common and attributed to longer LOS (38% ≥ 2 surgical interventions). Barrier height at the scene of injury was markedly associated with increased RR for ISS > 15. Tibial plafond fracture and spine injury were markedly associated with longer LOS, greater ISS, and ISS > 15. Extremity and pelvic injuries constituted the majority with 67.8% of injuries in 97.3% of patients and 91.0% of all surgical interventions. Comorbidities and COVID-19 markedly increased hospital LOS and affected surgical staging and delayed discharge. There was difficulty associated with language barriers, unpredictable access to care, and suboptimal support systems, reflected by only a 12% affiliated clinic follow-up rate. Opportunity abounds for improving the care of these patients. Recommendations include access to early appropriate care and transitions to locations with surgical care.

## Supplementary Material
