# Supplementary material for: Injuries Sustained With Falls From Height in Crossing the United States-Mexico Border at a Level I Trauma Center: A Prospective Cohort Study
Source: J Am Acad Orthop Surg Glob Res Rev. 2023 Jun 7;7(6):e23.00005. doi: 10.5435/JAAOSGlobal-D-23-00005 (PMC10249713; doi:10.5435/JAAOSGlobal-D-23-00005)
Supplement: Supplementary file 1 [file jagrr-7-e23.00005-s001.docx]

Supplemental Table 1: Comorbidities on presentation

| **Comorbidity** | **Total (N, %)** |
| --- | --- |
| Patients | 111 (24.7) |
| Tobacco/Marijuana | 37 (8.2) |
| COVID-19 | 31 (6.9) |
| Hypertension | 24 (5.3) |
| Diabetes Mellitus II | 15 (3.3) |
| Pregnancy | 8 (1.7) |
| Behavioral Health/Substance Abuse Disorder | 5 (1.1) |
| Paraplegia from Spinal Cord Injury | 2 (0.4) |
| Rhabdomyolysis | 2 (0.4) |
| *Other*  Asthma  Influenza  Osteogenesis Imperfecta  Surgical Site Infection on Arrival  Tuberculosis  Thyroid nodule  Diabetes Mellitus I | *7 (1.5)* 1 (0.2)  1 (0.2)  1 (0.2)  1 (0.2)  1 (0.2)  1 (0.2)  1 (0.2) |
| **Total** | **131 (29.2)** |

Supplemental Table 2: Upper extremity orthopaedic injuries

| **Injury** | **N** | **Open**  **(N, %)** | **Gustilo Anderson (N,%)** | | | **Operative**  **(N, %)** | **Location**  **(%)** | **Total***  **(%)** |
| --- | --- | --- | --- | --- | --- | --- | --- | --- |
|  |  |  | **1** | **2** | **3A** |  |  |  |
| Radial distal fx | 15 | 2 (13.3) | 1 (6.6) | 1 (6.6) | 0 (0) | 11 (73.3) | 14.4 | 2.1 |
| Hand laceration | 6 | 6 (100) | 0 (0) | 0 (0) | 0 (0) | 3 (50) | 5.8 | 0.8 |
| Scaphoid fx | 6 | 1 (16.6) | 0 (0) | 0 (0) | 0 (0) | 4 (66.6) | 5.8 | 0.8 |
| Elbow terrible triad | 5 | 1 (20) | 1 (20) | 0 (0) | 0 (0) | 5 (100) | 4.8 | 0.7 |
| Radial head fx | 5 | 1 (20) | 0 (0) | 1 (20) | 0 (0) | 3 (60) | 4.8 | 0.7 |
| Humeral distal fx | 4 | 3 (75) | 0 (0) | 2 (50) | 1 (25) | 4 (100) | 3.8 | 0.6 |
| Monteggia fx | 4 | 3 (75) | 3 (75) | 0 (0) | 0 (0) | 4 (100) | 3.8 | 0.6 |
| Perilunate dislocation | 4 | 0 (0) | 0 (0) | 0 (0) | 0 (0) | 4 (100) | 3.8 | 0.6 |
| Ulnar diaphyseal fx | 4 | 1 (25) | 1 (25) | 0 (0) | 0 (0) | 3 (75) | 3.8 | 0.6 |
| Ulnar styloid fx | 4 | 0 (0) | 0 (0) | 0 (0) | 0 (0) | 0 (0) | 3.8 | 0.6 |
| Finger fx | 3 | 1 (33.3) | 0 (0) | 0 (0) | 1 (33.3) | 2 (66.6) | 2.9 | 0.4 |
| Radial and ulnar distal fx | 3 | 3 (100) | 2 (66.6) | 1 (33.3) | 0 (0) | 3 (100) | 2.9 | 0.4 |
| Radial neck fx | 3 | 0 (0) | 0 (0) | 0 (0) | 0 (0) | 3 (100) | 2.9 | 0.4 |
| Shoulder dislocation | 3 | 0 (0) | 0 (0) | 0 (0) | 0 (0) | 0 (0) | 2.9 | 0.4 |
| Acute carpal  tunnel syndrome | 2 | 0 (0) | 0 (0) | 0 (0) | 0 (0) | 2 (100) | 1.9 | 0.3 |
| Arm laceration | 2 | 2 (100) | 0 (0) | 0 (0) | 0 (0) | 2 (100) | 1.9 | 0.3 |
| Clavicle fx | 2 | 0 (0) | 0 (0) | 0 (0) | 0 (0) | 0 (0) | 1.9 | 0.3 |
| Hand fx | 2 | 2 (100) | 0 (0) | 0 (0) | 2 (100) | 2 (100) | 1.9 | 0.3 |
| Pisiform fx | 2 | 0 (0) | 0 (0) | 0 (0) | 0 (0) | 0 (0) | 1.9 | 0.3 |
| Radius/ulna fx | 2 | 0 (0) | 0 (0) | 0 (0) | 0 (0) | 2 (100) | 1.9 | 0.3 |
| Scapula fx | 2 | 0 (0) | 0 (0) | 0 (0) | 0 (0) | 0 (0) | 1.9 | 0.3 |
| Triquetral fx | 2 | 0 (0) | 0 (0) | 0 (0) | 0 (0) | 2 (100) | 1.9 | 0.3 |
| Acromioclavicular  joint separation | 1 | 0 (0) | 0 (0) | 0 (0) | 0 (0) | 1 (100) | 1.0 | 0.1 |
| Coracoid fx | 1 | 0 (0) | 0 (0) | 0 (0) | 0 (0) | 0 (0) | 1.0 | 0.1 |
| Elbow dislocation | 1 | 0 (0) | 0 (0) | 0 (0) | 0 (0) | 1 (100) | 1.0 | 0.1 |
| Finger laceration | 1 | 1 (100) | 0 (0) | 0 (0) | 0 (0) | 1 (100) | 1.0 | 0.1 |
| Finger soft  tissue avulsion | 1 | 1 (100) | 0 (0) | 0 (0) | 0 (0) | 1 (100) | 1.0 | 0.1 |
| Finger traumatic  amputation | 1 | 1 (100) | 1 (100) | 0 (0) | 0 (0) | 1 (100) | 1.0 | 0.1 |
| Forearm traumatic  compartment syndrome | 1 | 1 (100) | 0 (0) | 0 (0) | 0 (0) | 0 (0) | 1.0 | 0.1 |
| Hand burn | 1 | 1 (100) | 0 (0) | 0 (0) | 0 (0) | 0 (0) | 1.0 | 0.1 |
| Hand degloving | 1 | 0 (0) | 0 (0) | 0 (0) | 0 (0) | 1 (100) | 1.0 | 0.1 |
| Hand ligament injury | 1 | 0 (0) | 0 (0) | 0 (0) | 0 (0) | 0 (0) | 1.0 | 0.1 |
| Humeral diaphysis fx | 1 | 0 (0) | 0 (0) | 0 (0) | 0 (0) | 1 (100) | 1.0 | 0.1 |
| Humeral greater  tuberosity fx | 1 | 0 (0) | 0 (0) | 0 (0) | 0 (0) | 0 (0) | 1.0 | 0.1 |
| Olecranon fx | 1 | 0 (0) | 0 (0) | 0 (0) | 0 (0) | 1 (100) | 1.0 | 0.1 |
| Proximal humerus fx | 1 | 0 (0) | 0 (0) | 0 (0) | 0 (0) | 1 (100) | 1.0 | 0.1 |
| Radial head/neck fx | 1 | 0 (0) | 0 (0) | 0 (0) | 0 (0) | 1 (100) | 1.0 | 0.1 |
| Radial nerve injury | 1 | 0 (0) | 0 (0) | 0 (0) | 0 (0) | 1 (100) | 1.0 | 0.1 |
| Radial nerve  laceration | 1 | 1 (100) | 0 (0) | 0 (0) | 0 (0) | 1 (100) | 1.0 | 0.1 |
| Radius fx | 1 | 0 (0) | 0 (0) | 0 (0) | 0 (0) | 1 (100) | 1.0 | 0.1 |
| Scapholunate  ligament injury | 1 | 0 (0) | 0 (0) | 0 (0) | 0 (0) | 1 (100) | 1.0 | 0.1 |
| Ulnar artery  thrombosis | 1 | 1 (100) | 1 (100) | 0 (0) | 0 (0) | 0 (0) | 1.0 | 0.1 |
| **Total** | **105** | **33**  **(31.7)** | **10**  **(9.6)** | **5**  **(4.8)** | **4**  **(3.8)** | **41**  **(39.4)** | **100** | **14.4** |
| **Median (IQR)** | **2 (2)** | **0 (1)**  **0 (100)** | **0 (0)**  **0 (0)** | **0 (0)**  **0 (0)** | **0 (0)**  **0 (0)** | **1 (3)**  **100 (100)** | **1.9 (2)** | **0.3 (0.3)** |
| **Range** | **1-15** | **0-6**  **(0-100)** | **0-3**  **(0-100)** | **0-2**  **(0-50)** | **0-2**  **(0-100)** | **0-11**  **(0-100)** | **1-14** | **0.1-2.1** |

IQR: interquartile range; fx: fracture

*Total extremity and pelvic ring and acetabular injuries

Supplemental Table 3: Lower extremity orthopaedic injuries

| **Injury** | **N** | **Open**  **(N, %)** | **Gustilo Anderson (N,%)** | | | | | | **Operative**  **(N, %)** | **Location**  **(%)** | **Total***  **(%)** |
| --- | --- | --- | --- | --- | --- | --- | --- | --- | --- | --- | --- |
|  |  |  | **1** | 2 | 3 | **3A** | **3B** | **3C** |  |  |  |
| Tibial plafond fx | 137 | 38 (27.7) | 7 (5.1) | 12 (8.7) | 18 (13.1) | 15 (10.9) | 2 (1.4) | 1 (0.7) | 137 (100) | 25.0 | 19.0 |
| Ankle fx | 73 | 7 (9.5) | 0 (0) | 3 (4.1) | 4 (5.4) | 4 (5.4) | 0 (0) | 0 (0) | 62 (84.9) | 13.3 | 10.1 |
| Calcaneus fx | 62 | 5 (8) | 2 (3.2) | 2 (3.2) | 1 (1.6) | 1 (1.6) | 0 (0) | 0 (0) | 15 (24.1) | 11.3 | 8.6 |
| Tibial diaphysis fx | 51 | 26 (50.9) | 4 (7.8) | 18 (35.2) | 3 (5.8) | 3 (5.8) | 0 (0) | 0 (0) | 51 (100) | 9.3 | 7.1 |
| Talus fx | 47 | 6 (12.7) | 1 (2.1) | 4 (8.5) | 1 (2.1) | 1 (2.1) | 0 (0) | 0 (0) | 23 (48.9) | 8.6 | 6.5 |
| Tibial plateau fx | 36 | 5 (13.8) | 2 (5.5) | 2 (5.5) | 1 (2.7) | 1 (2.7) | 0 (0) | 0 (0) | 33 (91.6) | 6.6 | 5.0 |
| Femoral proximal fx | 24 | 1 (4.1) | 0 (0) | 0 (0) | 1 (4.1) | 1 (4.1) | 0 (0) | 0 (0) | 22 (91.6) | 4.4 | 3.3 |
| Patella fx | 20 | 8 (40) | 3 (15) | 3 (15) | 2 (10) | 2 (10) | 0 (0) | 0 (0) | 18 (90) | 3.6 | 2.8 |
| Midfoot fx | 19 | 0 (0) | 0 (0) | 0 (0) | 0 (0) | 0 (0) | 0 (0) | 0 (0) | 10 (52.6) | 3.5 | 2.6 |
| Femoral diaphysis fx | 13 | 1 (7.6) | 0 (0) | 0 (0) | 1 (7.6) | 1 (7.6) | 0 (0) | 0 (0) | 13 (100) | 2.4 | 1.8 |
| Forefoot fx | 9 | 0 (0) | 0 (0) | 0 (0) | 0 (0) | 0 (0) | 0 (0) | 0 (0) | 0 (0) | 1.6 | 1.2 |
| Leg laceration | 8 | 8 (100) | 0 (0) | 0 (0) | 0 (0) | 0 (0) | 0 (0) | 0 (0) | 5 (62.5) | 1.5 | 1.1 |
| Knee traumatic  arthrotomy | 7 | 7 (100) | 0 (0) | 0 (0) | 0 (0) | 0 (0) | 0 (0) | 0 (0) | 7 (100) | 1.3 | 1.0 |
| Knee laceration | 5 | 5 (100) | 0 (0) | 0 (0) | 0 (0) | 0 (0) | 0 (0) | 0 (0) | 2 (40) | 0.9 | 0.7 |
| Femoral distal fx | 5 | 2 (40) | 0 (0) | 0 (0) | 2 (40) | 2 (40) | 0 (0) | 0 (0) | 5 (100) | 0.9 | 0.7 |
| Knee ligament injury | 4 | 0 (0) | 0 (0) | 0 (0) | 0 (0) | 0 (0) | 0 (0) | 0 (0) | 3 (75) | 0.7 | 0.6 |
| Knee dislocation | 3 | 0 (0) | 0 (0) | 0 (0) | 0 (0) | 0 (0) | 0 (0) | 0 (0) | 1 (33.3) | 0.5 | 0.4 |
| Proximal fibula fx | 3 | 0 (0) | 0 (0) | 0 (0) | 0 (0) | 0 (0) | 0 (0) | 0 (0) | 0 (0) | 0.5 | 0.4 |
| Tibiotalar  dislocation | 3 | 2 (66.6) | 0 (0) | 1 (33.3) | 0 (0) | 0 (0) | 0 (0) | 0 (0) | 3 (100) | 0.5 | 0.4 |
| Ankle traumatic  arthrotomy | 2 | 2 (100) | 0 (0) | 0 (0) | 0 (0) | 0 (0) | 0 (0) | 0 (0) | 2 (100) | 0.4 | 0.3 |
| Anterior tibial artery  occlusion | 2 | 1 (50) | 0 (0) | 0 (0) | 0 (0) | 0 (0) | 0 (0) | 0 (0) | 1 (50) | 0.4 | 0.3 |
| Navicular fx | 2 | 0 (0) | 0 (0) | 0 (0) | 0 (0) | 0 (0) | 0 (0) | 0 (0) | 1 (50) | 0.4 | 0.3 |
| Deep peroneal  nerve injury | 2 | 0 (0) | 0 (0) | 0 (0) | 0 (0) | 0 (0) | 0 (0) | 0 (0) | 0 (0) | 0.4 | 0.3 |
| Ankle sprain | 1 | 0 (0) | 0 (0) | 0 (0) | 0 (0) | 0 (0) | 0 (0) | 0 (0) | 0 (0) | 0.2 | 0.1 |
|  |  |  |  |  |  |  |  |  |  |  |  |
| Leg degloving | 1 | 1 (100) | 0 (0) | 0 (0) | 0 (0) | 0 (0) | 0 (0) | 0 (0) | 1 (100) | 0.2 | 0.1 |
| Leg hematoma | 1 | 0 (0) | 0 (0) | 0 (0) | 0 (0) | 0 (0) | 0 (0) | 0 (0) | 0 (0) | 0.2 | 0.1 |
| Patellar tendon  laceration | 1 | 1 (100) | 0 (0) | 0 (0) | 0 (0) | 0 (0) | 0 (0) | 0 (0) | 1 (100) | 0.2 | 0.1 |
| Patellar tendon  rupture | 1 | 1 (100) | 0 (0) | 0 (0) | 0 (0) | 0 (0) | 0 (0) | 0 (0) | 1 (100) | 0.2 | 0.1 |
| Popliteal artery  laceration | 1 | 0 (0) | 0 (0) | 0 (0) | 0 (0) | 0 (0) | 0 (0) | 0 (0) | 1 (100) | 0.2 | 0.1 |
| Popliteal artery  occlusion | 1 | 0 (0) | 0 (0) | 0 (0) | 0 (0) | 0 (0) | 0 (0) | 0 (0) | 1 (100) | 0.2 | 0.1 |
| Posterior tibial  tendon laceration | 1 | 0 (0) | 0 (0) | 0 (0) | 0 (0) | 0 (0) | 0 (0) | 0 (0) | 1 (100) | 0.2 | 0.1 |
| Soft tissue infection | 1 | 1 (100) | 0 (0) | 0 (0) | 1 (100) | 1 (100) | 0 (0) | 0 (0) | 1 (100) | 0.2 | 0.1 |
| Talonavicular  dislocation | 1 | 0 (0) | 0 (0) | 0 (0) | 0 (0) | 0 (0) | 0 (0) | 0 (0) | 0 (0) | 0.2 | 0.1 |
| Thigh laceration | 1 | 1 (100) | 0 (0) | 0 (0) | 0 (0) | 0 (0) | 0 (0) | 0 (0) | 1 (100) | 0.2 | 0.1 |
| Hallux fx | 1 | 0 (0) | 0 (0) | 0 (0) | 0 (0) | 0 (0) | 0 (0) | 0 (0) | 1 (100) | 0.2 | 0.1 |
| **Total** | **549** | **128**  **(23.3)** | **19**  **(3.5)** | **45**  **(8.2)** | **34**  **(6.2)** | **32**  **(5.8)** | **2**  **(0.36)** | **1**  **(0.18)** | **424**  **(77.1)** | **100** | **76.1** |
| **Median (IQR)** | **3 (13.5)** | **1 (5)**  **8.8 (75)** | **0 (0)**  **0 (0)** | **0 (0)**  **0 (0)** | **0 (1)**  **0 (2.3)** | **0 (1)**  **0 (2.3)** | **0 (0)**  **0 (0)** | **0 (0)**  **0 (0)** | **1**  **(9.8)** | **0.5**  **(2.5)** | **0.4**  **(1.9)** |
| **Range** | **1-137** | **0-37**  **(0-100)** | **0-7**  **(0-15)** | **0-18**  **(0-35)** | **0-17**  **(0-100)** | **0-15**  **(0-100)** | **0-2**  **(0-1.5)** | **0-1**  **(0-0.7)** | **0-137**  **(0-100)** | **0.2-25** | **0.14-18.9** |

IQR: interquartile range; fx: fracture

*Total extremity and pelvic ring and acetabular injuries

Supplemental Table 4: Pelvic ring and acetabular orthopaedic injuries

| **Injury** | **Count** | **Open**  **(N, %)** | **Operative**  **(N, %)** | **Location**  **(%)** | **Total**  **(%)*** |
| --- | --- | --- | --- | --- | --- |
| Pelvic ring fx | 42 | 0 (0) | 21 (50.0) | 60.9 | 5.8 |
| Coccyx fx | 21 | 0 (0) | 2 (9.5) | 30.4 | 2.9 |
| Acetabular fx | 6 | 0 (0) | 5 (83.3) | 8.7 | 0.8 |
| **Total** | 69 | 0 | 28  (40.6) | 100 | 10 |
| **Median (IQR)** | 13.5 (21) | 0 (0) | 3.5 (7)  66 (49) | 19.6 (30.8) | 1.9 (2.9) |
| **Range** | 6-42 | 0 | 2-21  (9.5-100) | 1.4-59.4 | 0.8-5.8 |

IQR: interquartile range; fx: fracture

*Total extremity and pelvic ring and acetabular injuries

Supplemental Table 5: Pelvic ring injuries by Young-Burgess and sacral fracture by Denis classifications

| **Pelvic Ring Injury** | **N (%)** | **Operative (%)** |
| --- | --- | --- |
| LC I, zone 1 sacral incomplete | 15 (35.7) | 2 (13) |
| LC I, zone 2 sacral unstable | 7 (16.7) | 3 (43) |
| LC I, zone 1 sacral complete | 3 (7.1) | 1 (33) |
| Vertical shear, zone 3 sacral, U-type | 3 (7.1) | 3 (100) |
| LC I, zone 2 sacral stable | 2 (4.8) | 2 (100) |
| LC II | 2 (4.8) | 2 (100) |
| LC II, zone 2 sacral unstable | 2 (4.8) | 2 (100) |
| Vertical shear, zone 2 sacral unstable | 2 (4.8) | 2 (100) |
| APC I | 1 (2.4) | 0 (0) |
| APC II | 1 (2.4) | 0 (0) |
| APC II, zone 2 sacral unstable | 1 (2.4) | 1 (100) |
| LC I, zone 2 sacral unstable | 1 (2.4) | 1 (100) |
| LC I, zone 3 sacral, H-type | 1 (2.4) | 1 (100) |
| LC I, zone 3 sacral, U-type | 1 (2.4) | 1 (100) |
| **Total** | **42 (100)** | **21 (50)** |

APC: anterior posterior compression; LC: lateral compression

Supplemental Table 6: Acetabular fractures

| **Acetabular Fracture** | **N (%)** | **Operative (%)** |
| --- | --- | --- |
| Both Column | 2 (33.3) | 2 (1000 |
| Transverse + Posterior Wall | 1 (16.7) | 1 (100) |
| Transverse | 1 (16.7) | 1 (100) |
| Posterior Wall | 1 (16.7) | 0 90) |
| Posterior Column | 1 (16.7) | 1 (100) |
| **Total** | **6 (100)** | **5 (83.3)** |

Supplemental Table 7: Spine injury levels

| **Spine Injury Level** | **Total Spine Injuries (N, %)** | **Operative** | |
| --- | --- | --- | --- |
|  |  | **Spine Injuries (N, %)** | **Level (%)** |
| C | 2 (1.3) | 1 (2.5) | 50.0 |
| T | 12 (7.8) | 4 (10.2) | 33.3 |
| TL | 87 (57.2) | 30 (76.9) | 34.5 |
| L | 51 (33.5) | 4 (10.2) | 7.8 |
| **Total** | **152** | **39 (25.6)** | **-** |
| **Median (IQR)** | **31.5 (51)**  **20.7% (33)** | **4.0 (7)**  **10.3% (19%)** | **33.9 (11)** |

C: cervical (C1-C7); IQR: interquartile range; L: lumbar (L3-L5): T: thoracic (T1-T9); TL: thoracolumbar (T10-L2)

Supplemental Table 8: Spine injury constellations by level for each patient with a spine injury

| **Spine Injury**  **Constellation** | **Total Spine Patients**  **(N, %)** | **Operative** | | |
| --- | --- | --- | --- | --- |
|  |  | **Spine**  **Patients (N, %)** | **Pattern (%)** | **All Patients (%)** |
| C/T | 1 (0.8) | 1 (0.8) | 100.0 | 0.2 |
| C/T/TL | 1 (0.8) | 0 (0) | 0.0 | 0.2 |
| L | 21 (17.9) | 4 (3.4) | 19.0 | 4.7 |
| T | 7 (5.9) | 3 (2.5) | 42.9 | 1.6 |
| T/L | 1 (0.8) | 0 (0) | 0.0 | 0.2 |
| T/TL | 2 (1.7) | 1 (0.8) | 50.0 | 0.4 |
| TL | 55 (47) | 23 (19.6) | 41.8 | 12.3 |
| TL/L | 29 (24.7) | 7 (5.9) | 24.1 | 6.5 |
| **Total** | **117**  **26.2% all patients** | **39 (33.3)** | **33.3** | **8.7** |
| **Median (IQR)** | **4.5 (20)**  **3.8% (19%)** | **2.0 (4)**  **1.7% (3%)** | **33.0 (30)** | **1.0 (5)** |

C: cervical; L: lumbar: T: thoracic; TL: thoracolumbar (T10-L2)

Supplemental Table 9: Spine injury morphologies

| **Morphology** | **Total Spine**  **Injuries (N, %)** | **Operative** | |
| --- | --- | --- | --- |
|  |  | **Spine Injuries (N,%)** | **Morphology (%)** |
| Spinous Process Fx | 7 (2.9) | 0 (0) | 0.0 |
| Transverse Process Fx | 43 (18.2) | 0 (0) | 0.0 |
| Compression Fx | 132 (55.9) | 1 (0.4) | 0.8 |
| Compression Fx >20% Height Loss | 9 (3.8) | 1 (0.4) | 11.1 |
| Burst Fx | 36 (15.2) | 30 (12.7) | 83.3 |
| Burst Fx with Paraplegia | 1 (0.4) | 1 (0.4) | 100.0 |
| Flexion-Distraction Fx | 5 (2.1) | 5 (2.1) | 100.0 |
| Flexion-Distraction Fx with Paraplegia | 1 (0.4) | 1 (0.4) | 100.0 |
| Acute Disc Herniation | 2 (0.8) | 0 (0) | 0.0 |
| **Total** | **236** | **39 (16.5)** | **-** |
| **Median (IQR)** | **7.0 (34)**  **3.0% (14%)** | **1.0 (1)**  **0.4% (0.4%)** | **11.1 (100)** |

Fx: fractureSupplemental Table 10: Head, neck, face, thorax, and abdominal injuries

| **Head and Neck** | | | **Face** | | | **Thorax** | | | **Abdomen** | | |
| --- | --- | --- | --- | --- | --- | --- | --- | --- | --- | --- | --- |
| **Injury** | **Region**  **(N, %)** | **Operative**  **(N, %)** | **Injury** | **Region**  **(N, %)** | **Operative**  **(N, %)** | **Injury** | **Region**  **(N, %)** | **Operative**  **(N, %)** | **Injury** | **Region**  **(N, %)** | **Operative**  **(N, %)** |
| TBI  ICH | 19  (48.7) | 4  (21) | Facial  laceration | 11  (44) | 1  (9) | Rib fx | 11  (37.9) | 0  (0) | Renal  laceration | 4  (28.5) | 0  (0) |
| Scalp  laceration | 6  (15.3) | 0  (0) | Facial fx | 9  (36) | 2  (22.2) | PTX | 5  (7.2) | 0  (0) | Hepatic  laceration | 3  (21.4) | 0  (0) |
| TBI  DAI | 5  (12.8) | 1  (20) | Orbital  blowout Periorbital  ecchymosis | 2  (8) | 0  (0) | Pulmonary  contusion,  bilateral | 4  (13.7) | 0  (0) | Bladder  injury | 1  (7.1) | 1  (100) |
| Skull fx | 4  (10.2) | 0  (0) | Eye  laceration | 1  (4) | 0  (0) | Rib fx  HTX | 1  (3.4) | 0  (0) | Cholecystitis | 1  (7.1) | 1  (100) |
| Scalp  hematoma | 2  (5.1) | 0  (0) | Periorbital  ecchymosis | 1  (4) | 0  (0) | Rib fx  PTX | 1  (3.4) | 0  (0) | Iliac artery  laceration | 1  (7.1) | 1  (100) |
| Skull fx  Scalp  laceration | 2  (5.1) | 1  (50) | Facial fx  Facial  laceration | 1  (4) | 0  (0) | Cardiac  arrest | 1  (3.4) | 0  (0) | Mesenteric  laceration | 1  (7.1) | 1  (100) |
| Thyroid  cancer | 1  (2.5) | 1  (100) |  | | | Rib fx  SC joint  dislocation | 1  (3.4) | 0  (0) | Spontaneous  abortion | 1  (7.1) | 1  (100) |
|  | | |  |  |  | Sternum  fx | 1  (3.4) | 0  (0) | Renal  laceration Adrenal  laceration | 1  (7.1) | 0  (0) |
|  |  |  |  |  |  | HTX | 1  (3.4) | 0  (0) | Splenic  laceration | 1  (7.1) | 0  (0) |
|  |  |  |  |  |  | PTX  HTX | 1  (3.4) | 0  (0) |  | | |
|  |  |  |  |  |  | Pulmonary  contusion | 1  (3.4) | 0  (0) |  |  |  |
|  |  |  |  |  |  | Sternum fx  Manubrium fx | 1  (3.4) | 0  (0) |  |  |  |
| Patients  (% all patients) | 30  (6.7) |  |  | 20  (4.5) |  |  | 19  (4.3) |  |  | 13  (2.9) |  |
| Total Injuries  (% Total*) | 39  (36.4) | 7  (46.7) |  | 25  (23.4) | 3  (2.8) |  | 29  (27.1) | 0  (0) |  | 14  (13.1) | 5  (4.7) |
| Median  (IQR) | 4  (3.5) | 1 (1)  20% (36%) |  | 1.5  (6.25) | 0 (0.75)  0% (6.8%) |  | 1  (0.75) | 0 (0)  0% (0%) |  | 1  (0) | 1 (1)  100 (100) |

DAI: diffuse axonal injury; fx: fracture; HTX: hemothorax; PTX: pneumothorax; SC: sternoclavicular; TBI: traumatic brain injury

*Head, neck, face, thorax, and abdominal injuries
